# Mineral Profiles Characteristics in Milk from Dairy Cows in Xinjiang, China, and Production Plan for Season-Dependent High-Calcium Milk Sources

**DOI:** 10.3390/foods14111841

**Published:** 2025-05-22

**Authors:** Li Liu, Zhuo Yang, Yongqing Li, Yikai Fan, Chu Chu, Haitong Wang, Ayihumaer Amantuer, Lijun Cao, Bo Hu, Zunongjiang Abula, Bo Zuo, Juncheng Huang, Shujun Zhang

**Affiliations:** 1 Key Laboratory of Agricultural Animal Genetics, Breeding and Reproduction of Ministry of Education, Huazhong Agricultural University, No. 1 Shizishan Street, Hongshan District, Wuhan 430070, China; liuli17509991115@webmail.hzau.edu.cn (L.L.); yang_zhuo@webmail.hzau.edu.cn (Z.Y.); liyongqing@webmail.hzau.edu.cn (Y.L.); fanyikai123@webmail.hzau.edu.cn (Y.F.); chu1999@webmail.hzau.edu.cn (C.C.); htw0411@webmail.hzau.edu.cn (H.W.); ahu-321@webmail.hzau.edu.cn (A.A.);; 2 Frontiers Science Center for Animal Breeding and Sustainable Production, Huazhong Agricultural University, Ministry of Education, Wuhan 430070, China; 3 Xinjiang Academy of Animal Science, No. 468, Ali Mountain Street, Shayibake District, Urumqi 830063, China; 15299475912@163.com (L.C.); 17509991115@163.com (J.H.)

**Keywords:** Xinjiang, China, milk minerals, secretion characteristics, native calcium milk sources, economic benefits

## Abstract

Mineral content is an important nutrient component in milk. At present, there is not much research and application on the ecological mineral profiles of milk, especially in the development and utilization of the dominant milk source in Xinjiang. This study uses a mid-infrared spectroscopy (MIRS) model to predict the mineral content of seven key minerals in milk, explores the secretion patterns and characteristics of mineral profiles in milk, provides production methods for the efficient utilization of high-calcium milk sources, and analyzes the possible economic benefits. The results indicate that the mineral content of milk in Xinjiang has advantages over that of other regions of China. Mineral profiles’ characteristics in milk are influenced by the parity, days of lactation, sampling season, calving season, and breast health status. Moreover, there are correlations between different minerals. Milk with higher calcium content also has elevated levels of other minerals and regular milk components (milk protein and milk fat). Therefore, such milk may serve as a reference for producing season-dependent high-calcium milk sources. If native calcium content above 1300 mg/kg, as identified in this study, was used to produce high-calcium pasteurized fresh milk and premium high-calcium pasteurized fresh milk, the dairy industry could see a significant increase in economic benefits. This study provides a foundation for the production of characteristic milk sources and diversified dairy products in Xinjiang. It also lays the groundwork for understanding the secretion patterns and mechanisms of minerals in milk.

## 1. Introduction

Xinjiang province, located in the northwest of China, covers a vast area of 1.6649 million square kilometers. It is a traditional pastoral region and one of China’s key milk source areas. Xinjiang boasts 770 million acres of usable pastureland, with a rich variety of high-quality forage grasses, providing ideal conditions for dairy farming. The region’s dry climate and geographical isolation effectively reduce the risk of disease transmission, while its natural environment, free from industrial pollution, and clean water sources from glacial meltwater and deep underground aquifers contribute to the superior quality of Xinjiang’s milk. Known for its natural purity, rich nutritional content, and smooth, creamy taste, Xinjiang dairy products are favored by consumers and widely recognized on the market.

Minerals are essential nutrients in milk, playing a crucial role in the skeletal and neuromuscular development of both adults and infants [1], as well as in regulating fluid balance and maintaining cardiovascular homeostasis [2]. Minerals are equally vital for the health and productivity of dairy cows [3,4,5,6]. They are involved in physiological functions such as membrane transport and osmotic balance and are also integral components of energy molecules or coenzyme factors [7,8]. Furthermore, milk minerals are closely linked to the processing characteristics of dairy products, particularly calcium phosphate and calcium ions, which contribute to micelle stability and, in turn, affect the properties and storage quality of dairy products [9,10]. Therefore, understanding the mineral content characteristics and secretion patterns in milk is highly meaningful, not only for the health management of dairy cows but also for improving milk quality and optimizing dairy product processing [11]. Currently, high-mineral milk (such as high-calcium milk, zinc/iron-fortified milk, etc.) sold on the consumer market mainly comes from minerals by being added during processing rather than them being secreted by the cow itself. Therefore, ecological high-mineral milk sources are precious, both in terms of economic value and food health. On the one hand, it can avoid potential risks caused by exogenous addition, and on the other hand, its natural high-mineral characteristics also have significant advantages in simplifying the processing task.

Currently, some researchers have explored the relationship between feed type, farming environment, cow health status, and mineral metabolism [12,13,14,15]. However, studies on the secretion patterns and characteristics of minerals in dairy cow milk remain relatively scarce [16,17]. The complex procedures and high costs associated with national standard methods (GB5009.268-2016) for determining mineral content make it difficult to conduct related research on a large sample scale.

Mid-infrared spectroscopy (MIRS) can detect the vibration absorption of specific chemical bonds (such as C-H, O-H, N-H) in milk components and establish the quantitative relationship between spectral characteristics and component concentration combined with stoichiometric models to achieve composition prediction. In recent years, MIRS has gained widespread application in the fields of milk quality control and dairy cow performance measurement due to its non-destructive, accurate, and rapid batch detection capabilities [18,19]. Many researchers developed milk MIR models to predict traits related to milk quality; for example, they explored major fatty acids [20], amino acids [21], coagulation properties [22], hyperketonemia [23], and dry matter intake [24]. In addition, some studies have demonstrated the potential of MIRS in predicting milk mineral content [25,26]. However, building mineral content prediction models is difficult because of the workload and price of determining mineral and spectral data in large samples. Moreover, the application of mineral prediction models remains limited, mainly due to factors such as model prediction accuracy and intellectual property concerns. In particular, large-scale population application studies are still scarce. In addition, there are no relevant studies that consider the aspects of milk quality and economic performance according to different ecological high-calcium milk sources.

Therefore, this study aimed to (1) use the mineral model established in our laboratory with good predictive effect and a certain application value to predict the mineral content in dairy cow milk from the Xinjiang region of China and analyze the characteristics of these minerals; (2) investigate the influencing factors and variations in mineral profiles in milk from dairy cows; (3) use the sources of variation influencing the contents of minerals in milk to explore the production plan for calcium-rich milk sources and analyze the economic benefits of ecological high-calcium milk production.

This study seeks to deepen the understanding of milk mineral characteristics, support the optimization and development of distinctive milk sources, and provide data and practical guidance for enterprises engaged in differentiated dairy product processing, especially in the applications of ecological high-calcium milk sources.

## 2. Materials and Methods

### 2.1. Dairy Cows and Milk Sample Collection

The dairy cows were sourced from 13 large-scale dairy farms in the Xinjiang region of China, with a total of 16,944 cows and 59,643 milk samples collected. These samples represented three dairy cow breeds: Holstein, Brown Cattle, and Simmental. Over a period of 15 months from February 2023 to May 2024, milk samples were collected monthly from each cow in accordance with the Chinese technical specifications for Holstein cow production performance testing (NY/T1450-2007). After adding preservative (bronopol), the samples were transported to the Dairy Herd Improvement (DHI) laboratory for analysis. The FOSS Milkoscan FT+ spectrometer (Hillerød, Denmark) was used to measure conventional milk components (milk fat, protein, and lactose content), somatic cell count, and Fourier Transform Infrared Spectroscopy (MIRS) [27]. Outliers in spectral data were identified and removed by calculating the standardized Mahalanobis Distance (MD). For each milk sample, principal component analysis (PCA) was performed on the 875 absorbance values, and the average Mahalanobis Distance was determined from the scores of the first three principal components. Samples with MD > 5 were excluded as spectral outliers [28].

Prior to the instrument use, calibration and standardization were performed using standardized samples from the Chinese National Animal Husbandry Station’s laboratory. Additionally, relevant cow and production performance data were collected.

### 2.2. Selection of Valid Samples

In this study, the modeling process followed the approach suggested by Christophe et al. [29], which incorporated milk samples from multiple regions and breeds to enhance model generalization and improve stability during validation. However, as model generalization increases, data may be affected by variations in the operators, processing methods, and, particularly, differences in the brands and models of measurement instruments, leading to potential errors. Even with data standardization [30], discrepancies may arise due to variations in the standard materials used and the standardization methods applied.

To improve the prediction accuracy and stability of the model, this study established four criteria: 1. Consistent sampling time span: the modeling and prediction samples both covered milk samples collected monthly from February 2023 to May 2024. 2. Consistent sampling farms: the modeling and prediction samples were sourced from the same set of farms. 3. Consistent measurement methods: all conventional milk component data and mid-infrared spectroscopy (MIRS) data were measured using the same FOSS instrument and method. 4. Consistent data selection method: both modeling and prediction samples excluded outliers, with valid data defined as values within the range of the mean ±3 times the standard deviation. Therefore, under the premise of adhering to these four standards, regular calibration and stability tests were performed using standardized samples to ensure the accuracy and reliability of the instruments used in this study. From the samples collected in Section 2.1, valid records were retained for the following parameters: milk fat content ranging from 1.5% to 9.0%, milk protein content ranging from 1% to 7%, somatic cell count < 100,000/mL, and lactation days ranging from 5 to 365 days. A total of 59,065 valid records were retained.

### 2.3. Prediction of Mineral Content in Milk Samples

The laboratory previously developed a milk mineral prediction model. Using the new MCRF strategy to select characteristic spectra related to 10 minerals, combined with five spectral preprocessing methods (MSC, SNV, SG+1D, SG+2D, and none) and the PLSR algorithm, prediction models for the content of each mineral were constructed. The full spectrum without feature selection (ALL, 875 points after excluding water absorption regions) was used as a control. The prediction performance of these models built with the SNV preprocessing method, MCRF feature selection, and PLSR modeling algorithm performed best. The result is shown in Table 1. The validation set for five mineral models had an RPDp greater than 1.5 (with Mg and Zn exceeding 1.4), indicating moderate to high practical value, and could be subjected to trials within production performance testing [31]. Among them, the prediction model of Ca demonstrated strong performance, with a determination coefficient (R^2^) of 0.957, a root-mean-square error (RMSE) of 38.87 mg/kg, and a performance deviation ratio (RPD) of 4.85. According to Soyeurt et al. [20], equations with R^2^ > 0.95 can be used in the milk payment systems.

The mineral content in the milk was predicted using the models shown in Table 1, and data outside the range of the mean ±3 standard deviations were treated as outliers and excluded. The final retained dataset for prediction contained 53,956 samples.

### 2.4. Calibration of Predicted Mineral Content Values

A general linear mixed model was established using the lmertest package in R version 4.2.1 to calibrate the phenotypic data for the seven mineral contents and obtain the calibrated prediction values. The calibration formula was as follows:

$$y_{ijklmn}=\mu +C_{i}+P_{j}+D_{k}+T_{l}+S_{m}+{HTD}_{n}+e_{ijklmn}$$

where *μ* represents the mean of the trait prediction values; *C_i_* denotes the calving season effect, divided into 4 levels (*i* = spring, summer, autumn, winter); *P_j_* represents the parity effect, divided into 3 levels (*j* = 1, 2, ≥3); *D_k_* denotes the lactation period effect, with lactation days ranging from 5 to 365, grouped in 30-day intervals and divided into 12 levels (*k* = 1–12); *T_l_* represents the sampling quarter effect, divided into 4 levels (*l* = spring, summer, autumn, winter); *S_m_* is the somatic cell score effect, divided into 7 levels (*m* = −2, −1, 0, 1, 2, 3, 4, 5) [32]; *HTD_n_* denotes the individual-measurement-date random effect; *e_ijklmn_* represents the random residual effect. The calibration value used for each sample was the predicted milk composition value minus the mean of the related fixed effects.

### 2.5. Correlation Analysis of Conventional Milk Composition, Mineral Profiles, and Their Interrelationships

The Pearson correlation coefficients and *p*-values between the predicted mineral content, conventional milk composition, and milk production traits were calculated using the Himisc package in R version 4.2.1. The correlation matrix was visualized using the corrplot function. The formula for calculating the correlation was as follows [29]:

$$\rho_{x,y}=\frac{\sigma_{\left(x,y\right)}}{{SD}_{x}{SD}_{y}}$$

where *x* and *y* represent the correlated traits; *ρ* is the correlation coefficient; σ is the covariance between *x* and *y*; and *SD* are the standard deviations of *x* and *y*, respectively.

### 2.6. Economic Effect Analysis

Based on the calcium content standards of these commercially available products, the samples in this study were divided into three groups: fresh milk, high-calcium fresh milk, and high-end fresh milk. Since the samples were collected once a month and each sample represents the average milk production of the cow within a 30-day period, the daily milk yield of each sample was multiplied by 30 to estimate the monthly milk yield for each cow. Using the sample size and average monthly milk yield for each group, the total milk yield of the herd could be calculated. By factoring in the price differences between the products with varying calcium contents, the potential economic effects between the sample groups with different calcium levels could be estimated.

### 2.7. Statistical Analysis

All statistical analyses were performed using R software version 4.2.1. The sklearn library was used for outlier detection. Differences between the least squares’ means were tested using the Bonferroni multiple comparison post hoc test (*p* < 0.05).

## 3. Results and Discussion

### 3.1. Mineral Content of Milk in Xinjiang Region Measured Using National Standard Methods

The mineral content of seven minerals in milk from the Xinjiang region was measured according to national standard methods. Table 2 showed that compared to two major dairy production areas in China (regions A and B), the mineral content of calcium (Ca), magnesium (Mg), phosphorus (P), potassium (K), and sodium (Na) in Xinjiang milk was significantly higher. Specifically, the Ca content was 10.04% and 10.18% higher than that in regions A and B, respectively, and the K content was 33.04% and 14.72% higher than that in regions A and B. The trace elements zinc (Zn) and strontium (Sr) were also significantly higher in Xinjiang, with increases of 37.85% and 66.67% compared to region A.

Compared with the literature values [33] (1159 mg/kg for Ca, 98 mg/kg for Mg, 939 mg/kg for P, 1430 mg/kg for K, 344 mg/kg for Na, 4.1 mg/kg for Zn, and 0.42 mg/kg for Sr), the mineral contents of Ca, Mg, P, K, Na, Zn, and Sr in Xinjiang milk were significantly higher by 14.75%, 13.26%, 9.79%, 19.44%, 54.36%, 28.78%, and 102.38%, respectively. These results indicated that the distribution of mineral content in Xinjiang milk had its own unique advantages and characteristics.

### 3.2. Prediction of Mineral Profiles and Characteristics in Large-Scale Milk Samples Using the Model

To further explore the mineral profiles and characteristics of milk, this study used the mineral prediction model previously established in our laboratory to predict the mineral content of a large-scale milk sample from the Xinjiang region. The results are shown in Table 3. The average mineral contents for Ca, Mg, P, K, Na, Zn, and Sr were 1213.78 mg/kg, 116.63 mg/kg, 1077.25 mg/kg, 1674.38 mg/kg, 530.78 mg/kg, 5.19 mg/kg, and 0.90 mg/kg, respectively. Among these, the Mg and Na contents were comparable to the average values measured using the national standard method, while the P and Sr contents were slightly higher than those measured by the national standard method, and the other mineral contents were slightly lower.

The national standard method was primarily used to establish the mineral prediction model and included only 303 samples, accounting for just 0.56% of the total sample size (53,956 samples). Therefore, it was normal for the means of the two datasets to differ, which was consistent with findings from other research teams [19].

When comparing the mineral content of the large-scale predicted samples with those from regions A and B, as well as the values reported in the literature, the Xinjiang milk samples showed higher levels of Ca, Mg, P, K, and Na. This highlights the importance of further investigating the mineral content and characteristics of Xinjiang milk to fully leverage local specialty milk sources and develop unique dairy products.

### 3.3. Correlation Between Conventional Milk Components, Mineral Content, and Their Interrelationships

**Figure 1:**
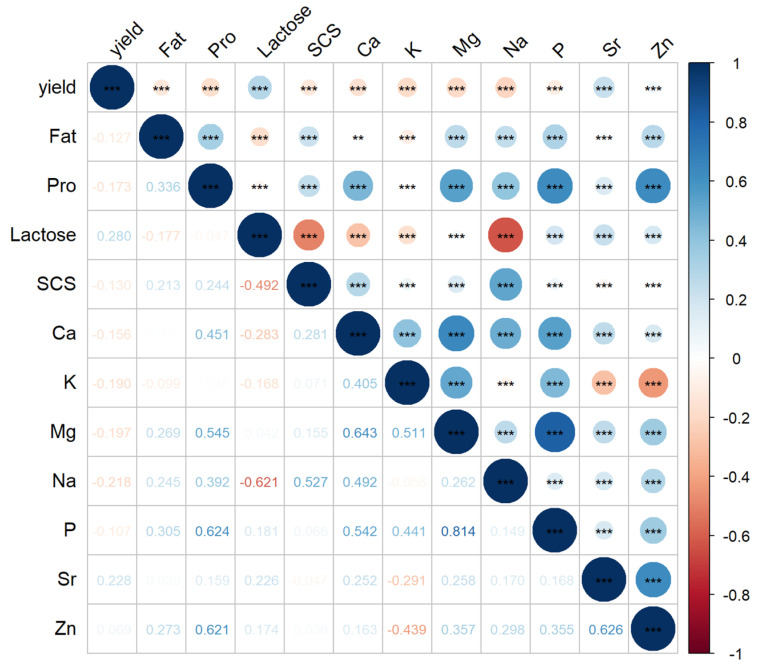
Map of Pearson correlations between milk minerals and major constituents. Ellipse colors represent the strength and the direction of the correlation, −1 to 0 (red to white to blue). ** *p* < 0.01; *** *p* < 0.001.

To explore the patterns of milk quality formation and variation, this study first analyzed the correlations among milk components. As shown in Figure 1, the fat content and protein content exhibited a moderate positive correlation of 0.335. Both fat and protein contents showed weak negative correlations with milk yield and lactose content ranging between −0.05 and −0.179 [34]. Fat and protein contents also had weak positive correlations with somatic cell score (SCS), with values of 0.213 and 0.244, respectively. Milk protein content showed moderate to strong positive correlations with the mineral contents of Ca, Mg, P, and Zn, ranging from 0.454 to 0.624. Fat content (Fat) had weak positive correlations with Mg, Na, P, and Zn, with values ranging from 0.244 to 0.304, and a weak negative correlation with K (−0.10). Lactose content showed weak positive correlations with Mg, P, Sr, and Zn (0.04–0.23), while it exhibited negative correlations with other minerals, ranging from −0.16 to −0.62. These results indicate that many minerals have stronger correlations with milk protein content than with fat or lactose content. As Stocco explained, Mg, Ca, and Zn are involved in the binding of casein micelles, and their variations are closely related to casein [35]. The correlation with fat content is indirect, arising from the relationship between fat and protein content [9].

Ca was strongly positively correlated with K, Mg, and P, with values ranging from 0.410 to 0.652. P showed a high positive correlation with Mg and K, with values of 0.814 and 0.441, respectively. Na exhibited a strong positive correlation with SCS (0.527). The correlations between K and Sr and Zn were −0.29 and −0.44, while K showed positive correlations with other mineral contents, consistent with findings in the literature [27,29,33].

### 3.4. Mineral Profiles Characteristics in Milk

To study the mineral profiles characteristics in milk under different secretion patterns, this research first analyzed the effects of environmental and physiological factors on the conventional milk components (fat, protein, lactose) and mineral content (Ca, Mg, P, K, Na, Zn, Sr). The results presented in Table 4 show that factors such as parity, lactation days, sampling season, and pregnancy season had a significant impact on the mineral content in milk (*p* < 0.05). The somatic cell score (SCS) also significantly influenced the mineral content in milk, except for Sr.

**Figure 2:**
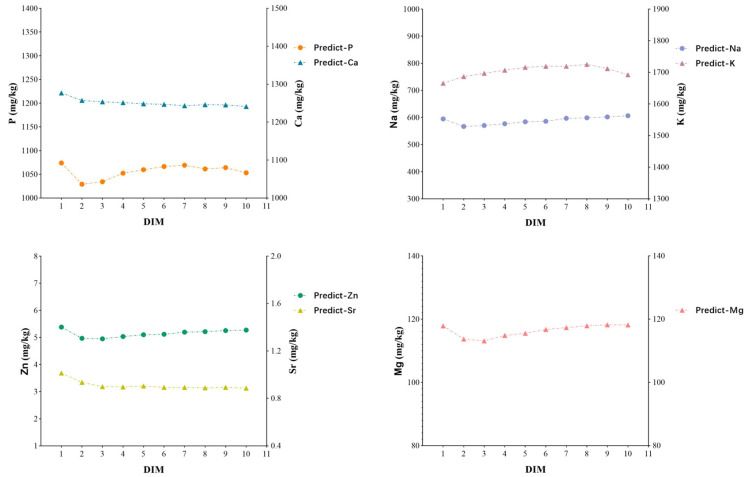
The trend in mineral content in the prediction set changing with lactation days.

Figure 2 illustrates that the concentrations of Ca, P, Na, Zn, and Sr significantly decreased during the second month of lactation, after which P, Na, and Zn gradually increased, while Ca and Sr stabilized. This variation may be related to the high metabolic load experienced by cows during early lactation, where the intake may not meet the nutritional demands of the body, leading to a reduction in milk components (such as fat, protein, and lactose) [36]. Additionally, the increased milk yield caused a dilution effect, which resulted in an overall decrease in mineral content. Moreover, K levels continuously rose from early lactation and began to decline slowly toward the end of lactation, while the trend for Mg was the opposite. This could be due to the elevated K levels disrupting the electrochemical balance required for Mg absorption [29,36,37].

Table 5 further illustrates the impact of parity on mineral content. The highest concentrations of P, K, Mg, and Zn were found in primiparous cows, while Ca, Na, and Sr peaked in cows with three or more parities. The variation in Ca content may be related to the incomplete skeletal development in cows during their first lactation, where more Ca is utilized for bone growth rather than milk production [29]. With an increase in parity, P content gradually decreased, which may be linked to a reduced phosphorus utilization efficiency in multiparous cows [38]. Additionally, the elevated Na levels and decreased K levels may be associated with an increased likelihood of illness in cows as the number of calvings rises [39].

The impact of sampling season on mineral content (Table 5) shows that the highest concentrations of P, K, and Mg were found in winter milk, while Zn, Sr, and Na concentrations were highest in autumn. In contrast, nearly all mineral contents (except K) were lowest during the summer. This phenomenon could be attributed to changes in dietary structure and temperature fluctuations [40]. Natural pasture affects the ion balance of feed, which in turn alters the mineral levels in the cows’ bodies [41]. Moreover, heat stress caused by high temperatures reduces the cows’ appetite, which affects nutrient intake and milk composition [7].

This study found that winter calving cows had the highest Ca content in their milk, which is consistent with findings in the literature [21]. However, P, Mg, K, and Na contents were higher in the milk of cows that calved in autumn, and Zn and Sr showed a significant advantage in milk from summer calving cows. This could be due to higher mineral content in the supplemental feed provided during the autumn and winter, and the fact that cows’ feed utilization efficiency is higher during the colder months, thus ensuring better mineral reserves [15].

**Figure 3:**
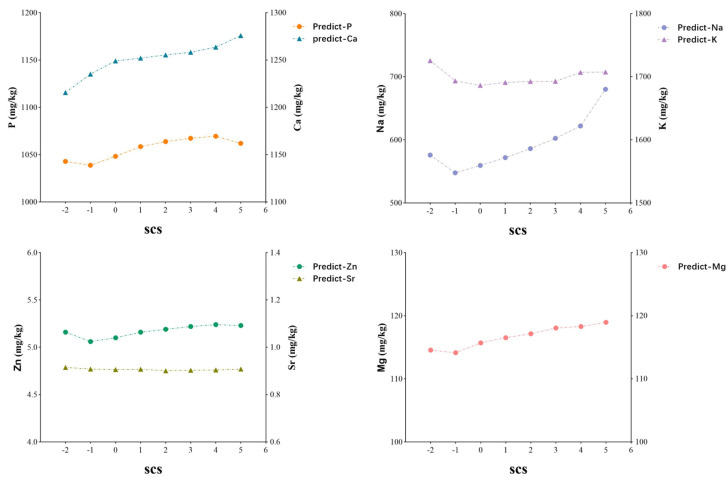
The trend in mineral content in milk samples from the prediction set with changes in somatic cell count.

As SCS increased (Figure 3), the concentrations of Ca, P, Na, Zn, and Mg generally rose. When SCS > 4, P content significantly decreased, while Ca, Mg, and Na levels increased, with Na showing a particularly significant change. SCS > 4 (somatic cell count > 200,000) is considered an indicator of subclinical mastitis in cows, and minerals are involved in osmotic pressure regulation caused by inflammation [42].

In conclusion, the mineral profiles of milk are influenced not only by feeding conditions and environmental factors but are also closely related to genetic factors and the physiological state of the cows [13], including their health status [35,40,43].

### 3.5. Production Plan for High-Calcium Milk from Season-Dependent Mineral Sources

Based on the characteristics of mineral profiles in milk, influencing factors, and secretion patterns, the results indicated that milk samples with a high Ca content (1200 mg/kg) primarily came from cows with three or more parities, calving in winter, in early lactation, and sampled in spring. As shown in Table 6, these milk samples had Ca content exceeding 1200 mg/kg, with fat content greater than 4.0% and protein content exceeding 3.43%, demonstrating overall higher milk quality. Notably, milk sampled in spring showed a significant advantage in Ca content, while milk from the early lactation stage exhibited the highest levels of fat, protein, and total solids, with highly significant differences. Therefore, milk from cows with three or more parities, calving in winter, in early lactation, and with an SCS of less than four is more suitable as a reference for producing high-Ca milk from season-dependent sources.

As shown in Figure 2, the Ca content in milk was positively correlated with other components (K, Mg, P). Table 7 shows that milk samples with Ca content greater than 1300 mg/kg had significantly higher levels of minerals, milk fat, protein, and total solids compared to milk samples with Ca content less than 1200 mg/kg. Specifically, the concentrations of Ca, K, Na, Mg, P, milk fat, protein, and total solids increased by 32.86%, 17.26%, 1.89%, 6.81%, 5.02%, 10.59%, 1.44%, and 0.60%, respectively. These results indicate that an increase in calcium content in milk can promote the enrichment of these substances. Therefore, milk from high-calcium sources not only provides a viable production plan for high-calcium milk but also offers important reference guidelines for producing milk products enriched with other minerals, as well as milk fat, protein, and total solids.

### 3.6. Economic Benefit Analysis of Dairy Products Enriched with Minerals (Ca)

Currently, in China, large dairy enterprises produce a variety of pasteurized milk products with different calcium contents. Among these, the eco-friendly high-calcium pasteurized fresh milk (high-calcium fresh milk, Ca content 1200 mg/L, fat content 4.2%, protein content 3.6%) is priced at 24.9 RMB/L; the eco-friendly high-end high-calcium pasteurized fresh milk (high-end fresh milk, Ca content 1300 mg/L, fat content 4.6%, protein content 4.0%) is priced at 32.9 RMB/L. Both of these are priced 8.2 RMB/L and 16.2 RMB/L higher, respectively, than the regular pasteurized fresh milk (fresh milk, Ca content 1100 mg/L, fat content 3.7%, protein content 3.1%), priced at 16.7 RMB/L.

According to the National Food Safety Standard’s *General Principles for Nutrition Labeling of Pre-packaged Foods* (GB 28050-2011) and the standards for popular ecological calcium products on the market, the Ca content, milk fat, and protein percentages for 6816 milk samples in this study were 1200 mg/kg, 4.14%, and 3.47%, respectively. For 19,580 other milk samples, these values were 1300 mg/kg, 4.17%, and 3.51%, which either met or closely aligned with the quality requirements for two types of high-calcium milk: ecological high-calcium pasteurized fresh milk (high-calcium fresh milk, 1200 mg/kg, 4.2% fat, 3.6% protein) and ecological high-calcium premium pasteurized fresh milk (premium fresh milk, 1300 mg/kg, 4.6% fat, 4.0% protein).

Although the Ca content of these samples met the relevant standards, the fat and protein percentages (4.14% and 3.47%; 4.17% and 3.51%) were slightly lower than those of the high-calcium fresh milk and premium fresh milk (4.2% and 3.6%, 4.6% and 4.0%, respectively). Therefore, assuming that the contribution of calcium content to dairy product pricing was 30%, and considering that the prices of high-calcium fresh milk and premium fresh milk were higher than regular fresh milk by 8.2 RMB/kg and 16.2 RMB/kg, respectively, this study estimated the following economic benefits:

If the 6816 milk samples with a calcium content of 1200 mg/kg in this study were processed into high-calcium fresh milk, the additional profit compared to regular fresh milk would be as follows: 6816 heads × 28.23 kg/day/head (average daily milk yield) × 30 days (1 month) × 8.2 RMB/kg (price difference) × 30% (contribution rate) = 14.2 million RMB.

Similarly, if the 19,580 milk samples with calcium content exceeding 1300 mg/kg were processed into premium fresh milk, the additional profit would be as follows: 77.76 million RMB (19,580 heads × 27.24 kg/day/head × 30 days × 16.2 RMB/kg × 30%). In total, the production of these two types of high-calcium milk (high-calcium fresh milk and premium fresh milk) would generate an additional profit of approximately 91.96 million RMB.

A further analysis considering the 15-month collection and testing period for the samples used in this study showed that the monthly economic benefit could be calculated as:

91.96 million RMB/15 months = 6.13 million RMB per month.

This benefit covers the entire industry chain, including dairy farming, dairy processing, and market sales. Assuming revenue shares for dairy farming, dairy processing, and milk sales of 20%, 40%, and 40%, respectively, the monthly added economic benefit for dairy farming would be 6.13 million RMB × 20% = 1.22 million RMB/month; for dairy processing and milk sales, the profit increase would be 6.13 million RMB × 40% = 2.45 million RMB/month each.

Therefore, compared to the production of regular pasteurized fresh milk, the production of ecological high-calcium pasteurized fresh milk and ecological high-calcium premium pasteurized fresh milk can significantly increase economic benefits, driving economic growth across the entire industry chain. This has a particularly positive economic impact on the dairy farming, dairy processing, and milk sales sectors.

### 3.7. Prospects and Limitation

The data utilized in this study were derived from predicted values based on the reliability assessment of the predictive model. During preliminary model construction, the prediction model for Ca demonstrated robust performance (R^2^ > 0.95) and met the application standards. A comparative analysis of the test and prediction datasets (Supplementary Figure S1) revealed highly congruent variation patterns with influencing factors and indicated that the predicted results were consistent with the variation rule of the tested values. However, discrepancies were observed at some loci, potentially attributable to the limited sample size of the test sets: 303 samples were randomly selected from the population sample (0.56% of the total dataset). Individual results have a greater effect on the mean values, especially in small sample sets [19,29]. Consequently, the prediction set characterized by large-scale data exhibited a smoother trend curve relative to the test set. This phenomenon may account for localized discrepancies and represents a limiting factor in further enhancing model efficacy.

Furthermore, this investigation prioritized the analysis of Ca variation patterns in conjunction with economic benefit evaluation, aiming to provide enterprises with a data-driven perspective for differentiation in product strategies. Environmental covariates of the same feeding method and genetic variabilities across cultivars were excluded from the analytical framework.

To address current limitations, future efforts will focus on 1. optimizing sampling strategy to improve test set generalizability [44]; 2. establishing cross-regional and cross-cultivar collaborative external reference populations to expand validation sample sizes, further validating the reliability of the model’s predicted values; and 3. provide detailed and accurate guidance for enterprise production.

## 4. Conclusions

The mineral content in milk from the Xinjiang region was relatively high, with approximately 50% of milk samples having a calcium content exceeding 1200 mg/kg. The mineral profiles in milk were significantly influenced by factors such as parity, sampling season, lactation days, calving season, and somatic cell score. The study found that milk in the early stages of lactation generally contained higher mineral levels, while the mineral content in milk during the summer was significantly lower. Additionally, cows calving in winter tended to produce milk with higher calcium content. Based on these findings, a preliminary reference production plan for high-calcium milk was proposed: healthy cows with at least three parities, calving in winter, and in the early stages of lactation.

High-calcium milk not only has higher levels of other minerals but also superior content of conventional milk components, significantly enhancing the overall quality and nutritional value of the milk. Therefore, producing fresh milk products with differentiated calcium content could generate an additional economic benefit of nearly RMB 6 million per month for the dairy farming, processing, and sales sectors.

This information contributes to a better understanding of the value of ecological high-calcium milk sources, laying the groundwork for its application in dairy product production. It also provides insights into achieving sustainable development and consumption upgrading. However, due to the limitations of the study sample and the complexity of multiple interacting factors, future research should be based on a broader population. Continued optimization in areas such as genetic breeding, feeding management, and processing technologies is necessary to promote the widespread production and application of native high-calcium milk.

## Figures and Tables

**Table 1:** Prediction performance of equations allowing us to predict the content (mg/kg of milk) of major minerals in bovine milk.

		Validation		
Mineral	LVs	R^2^p	RMSEp	RPDp
Ca	20	0.957	37.87	4.85
K	9	0.571	189.69	1.53
Mg	17	0.497	11.91	1.41
Na	12	0.660	67.97	1.71
P	16	0.745	67.91	1.98
Sr	11	0.663	0.14	1.72
Zn	11	0.529	0.77	1.46

LVs = latent variables; R^2^p = coefficient of determination of external validation; RMSEp = root-mean-square error of external validation; RPDp = residual predictive deviation.

**Table 2:** Descriptive statistics of mineral (mg/kg) concentrations measured in bovine colostrum via gold standards, *p* < 0.05.

Item	Xinjiang	Region A	Region B
Minerals, mg/kg			
Ca	1329.84 ± 193.37 ^a^	1208.49 ± 140.74 ^b^	1206.94 ± 125.49 ^b^
Mg	111.36 ± 17.59 ^a^	104.12 ± 11.31 ^a^	105.20 ± 10.25 ^a^
P	1031.31 ± 142.87 ^a^	1010.88 ± 119.34 ^a^	1017.65 ± 129.33 ^a^
K	1709.91 ± 484.93 ^a^	1285.22 ± 179.43 ^b^	1490.52 ± 142.71 ^b^
Na	531.06 ± 153.47 ^a^	311.15 ± 52.58 ^b^	470.93 ± 120.59 ^a^
Zn	5.28 ± 1.97 ^a^	3.83 ± 0.68 ^b^	5.86 ± 2.31 ^a^
Sr	0.85 ± 0.26 ^a^	0.51 ± 0.09 ^b^	0.92 ± 0.21 ^a^

For each column, mineral content with a, b and c superscripts are significantly different (*p* < 0.05).

**Table 3:** Descriptive statistics of conventional milk components (%), and mineral content (mg/kg) (N = 53,956).

Item	N	Mean	SD	Min	Max
Milk composition, %					
Fat	53,956	4.11	1.08	1.50	9.00
Protein	53,956	3.51	0.43	1.81	5.97
Lactose	53,956	5.17	0.27	2.88	5.93
Health					
SCC, 10^3^ mL^−1^	53,956	251.96	853	1	9974
SCS ^1^, score	53,956	1.93	1.76	−2	5
Macrominerals, mg/kg milk					
Ca	53,956	1213.78	177.32	704.00	1957.92
Mg	53,956	116.63	18.24	55.95	170.10
P	53,956	1077.25	159.20	554.03	1521.42
K	53,956	1674.38	264.27	784.93	3101.79
Na	53,956	530.78	122.14	125.24	1392.37
Trace minerals, mg/kg milk					
Zn	53,956	5.19	1.02	2.13	10.93
Sr	53,956	0.90	0.19	0.12	1.85

^1^ SCS = 3 + log_2_ (SCC/100,000).

**Table 4:** Analysis of variance for conventional milk components and mineral content (F-values and significance), * *p* < 0.05; ** *p* < 0.01; *** *p* < 0.001.

Item	Parity	DIM	Calving Season	Sample Season	SCS
Milk yield, kg/d	232.74 ***	515.29 ***	8.83 ***	770.89 ***	40.48 ***
Milk composition, %					
Fat	5.87 **	83.15 ***	48.93 ***	26.28 ***	88.28 ***
Protein	14.98 ***	370.69 ***	21.67 ***	1517.53 ***	191.30 ***
Lactose	236.06 ***	83.48 ***	2.86 *	903.67 ***	801.70 ***
Minerals, mg/kg					
Ca	34.12 ***	51.34 ***	76.65 ***	39,680.59 ***	90.71 ***
Mg	67.08 ***	58.20 ***	19.11 ***	2374.79 ***	40.68 ***
P	136.59 ***	49.63 ***	30.84 ***	2195.95 ***	32.72 ***
K	26.35 ***	28.74 ***	11.26 ***	4486.39 ***	8.54 ***
Na	103.71 ***	87.60 ***	5.99 ***	300.49 ***	895.34 ***
Trace minerals, mg/kg					
Zn	27.63 ***	124.88 ***	29.25 ***	4998.77 ***	24.46 ***
Sr	55.81 ***	245.83 ***	31.14 ***	3431.94 ***	1.84

**Table 5:** The least squares’ mean of the predicted milk minerals (mg/kg) predicted by the model under different influencing factors. *p* < 0.05. Means followed by different letters (within the factor) are significantly different.

	Parity			Sample Season				Calving Season			
Trait	1	2	≥3	Spring	Summer	Autumn	Winter	Spring	Summer	Autumn	Winter
Ca	1246.52 ^c^	1249.48 ^b^	1255.62 ^a^	1390.89 ^a^	1059.68 ^d^	1185.68 ^c^	1365.91 ^b^	1247.98 ^b^	1242.97 ^c^	1250.53 ^b^	1260.68 ^a^
P	1073.09 ^a^	1045.71 ^b^	1046.40 ^b^	1056.45 ^c^	958.71 ^d^	1068.67 ^b^	1136.44 ^a^	1051.66 ^b^	1051.57 ^b^	1066.41 ^a^	1050.63 ^b^
K	1711.78 ^a^	1691.76 ^b^	1694.94 ^b^	1753.95 ^b^	1613.82 ^c^	1570.78 ^d^	1859.40 ^a^	1703.46 ^a^	1692.76 ^b^	1706.63 ^a^	1695.10 ^b^
Na	583.62 ^c^	593.01 ^b^	602.75 ^a^	599.70 ^b^	573.61 ^d^	612.54 ^a^	586.66 ^c^	589.51 ^b^	594.56 ^a^	594.37 ^a^	594.06 ^a^
Mg	118.05 ^a^	115.74 ^b^	116.22 ^b^	114.42 ^c^	106.46 ^d^	118.96 ^b^	126.82 ^a^	116.21 ^b^	116.67 ^b^	117.66 ^a^	116.13 ^b^
Zn	5.21 ^a^	5.13 ^c^	5.16 ^b^	4.87 ^c^	4.89 ^c^	5.92 ^a^	5.00 ^b^	5.15 ^b^	5.23 ^a^	5.15 ^b^	5.15 ^b^
Sr	0.89 ^c^	0.90 ^b^	0.91 ^a^	0.83 ^d^	0.84 ^c^	1.02 ^a^	0.93 ^b^	0.89 ^c^	0.92 ^a^	0.91 ^b^	0.90 ^b^

**Table 6:** Differences in conventional milk components and calcium content under different influencing conditions.

Item	Ca	Fat	Pro	TS	MY
	mg/kg	%	%	%	kg
All herd average	1213.78 ± 177.32	4.11 ± 1.08	3.51 ± 0.43	13.27. ± 1.3	30.44 ± 14.13
Parity ≥ 3	1224.22 ± 185.97 ^c^	4.04 ± 1.11 ^e^	3.46 ± 0.42 ^b^	13.11 ± 1.32 ^d^	29.42 ± 16.18 ^c^
Calving season = winter	1249.85 ± 191.50 ^b^	4.14 ± 1.07 ^c^	3.46 ± 0.43 ^b^	13.25 ± 1.26 ^b c^	28.23 ± 13.07 ^d^
DIM ≤ 35	1226.25 ± 190.37 ^c^	4.40 ± 1.18 ^a^	3.43 ± 0.44 ^c^	13.51 ± 1.38 ^a^	32.11 ± 14.78 ^a^
Sample season = spring	1376.61 ± 143.84 ^a^	4.26 ± 1.01 ^b^	3.36 ± 0.37 ^d^	13.24 ± 1.17 ^c^	26.97 ± 12.61 ^e^
SCS < 4	1209.99 ± 174.09 ^d^	4.09 ± 1.06 ^d^	3.50 ± 0.42 ^a^	13.27 ± 1.28 ^b^	30.732 ± 14.13 ^b^

For each column, number with a, b and c superscripts are significantly different (*p* < 0.05).

**Table 7:** Mineral Content, Conventional Milk Components, and Milk Yield in Different Calcium Content Sample Groups.

Item	Ca < 1200 (Fresh Milk Group)	1200 ≤ Ca < 1300 (High-Calcium Fresh Milk Group)	1300 ≤ Ca (High-End Fresh Milk Group)
	n = 28,140	n = 6816	n = 19,580
Minerals, mg/kg			
Ca	1065.01 ± 66.37 ^c^	1249.96 ± 29.66 ^b^	1415.00 ± 90.45 ^a^
K	1555.86 ± 192.51 ^c^	1732.81 ± 294.18 ^b^	1824.38 ± 259.35 ^a^
Na	528.00 ± 94.98 ^b^	521.60 ± 107.81 ^c^	537.97 ± 156.36 ^a^
Mg	113.77 ± 15.76 ^c^	114.36 ± 21.96 ^b^	121.52 ± 19.09 ^a^
P	1055.64 ± 157.39 ^c^	1076.15 ± 179.36 ^b^	1108.68 ± 148.80 ^a^
Zn	5.56 ± 0.84 ^a^	4.91 ± 1.12 ^b^	4.75 ± 1.01 ^c^
Sr	0.95 ± 0.18 ^a^	0.86 ± 0.18 ^b^	0.85 ± 0.19 ^c^
Milk composition, %			
Fat	4.06 ± 1.05 ^c^	4.14 ± 1.09 ^b^	4.17 ± 1.13 ^a^
Pro	3.46 ± 0.49 ^b^	3.47 ± 0.38 ^b^	3.51 ± 0.43 ^a^
TS	13.23 ± 1.28 ^b^	13.30 ± 1.44 ^a^	13.31 ± 2.57 ^a^
Milk yield, kg/d	33.21 ± 13.72 ^a^	28.23 ± 13.64 ^b^	27.24 ± 14.07 ^c^

For each column, number with a, b and c superscripts are significantly different (*p* < 0.05).

## Data Availability

The data presented in this study are available on request from the corresponding author.

## Supplementary Materials

The following supporting information can be downloaded at: https://www.mdpi.com/article/10.3390/foods14111841/s1, Figure S1: The trend of protein, fat, Ca, P contents in the prediction and tested set changing with lactation days.
