# Supplementary material for: Mineral Profiles Characteristics in Milk from Dairy Cows in Xinjiang, China, and Production Plan for Season-Dependent High-Calcium Milk Sources
Source: Foods. 2025 May 22;14(11):1841. doi: 10.3390/foods14111841 (PMC12154361; doi:10.3390/foods14111841)
Supplement: Supplementary file 1 [file foods-14-01841-s001.zip › foods-3501579-supplementary.pdf]

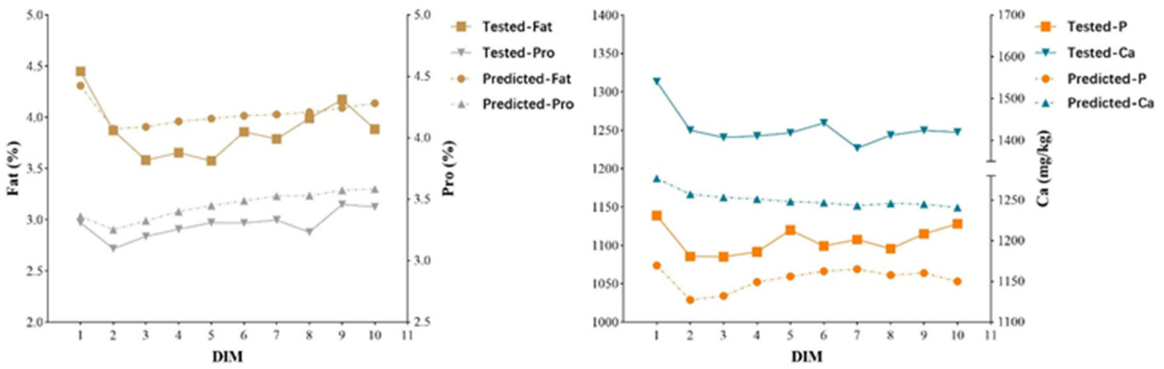


**Supplement Figure 1** The trend of protein, fat, Ca, P contents in the prediction and tested set changing with lactation days
